# Rectum separation in patients with cervical cancer for treatment planning in primary chemo-radiation

**DOI:** 10.1186/1748-717X-7-109

**Published:** 2012-07-12

**Authors:** Simone Marnitz, Volker Budach, Friederike Weißer, Elena Burova, Bernhard Gebauer, Filiberto Guiseppe Vercellino, Christhardt Köhler

**Affiliations:** 1 Department of Radiooncology, Charité University Medicine, Augustenburger Platz 1, 13353, Berlin, Germany; 2 Institute of Radiology, University Medicine, Augustenburger Platz 1, 13353, Berlin, Germany; 3 Campus Benjamin Franklin, Department of Gynecology Campus CBF, Charité University Medicine, Hindenburgdamm 30, 12200, Berlin, Germany; 4 Campus Mitte, Department of Gynecology, Charité University Medicine, Charitéplatz 1, 10117, Berlin, Germany

## Abstract

**Purpose:**

To proof feasibility of hydrogel application in patients with advanced cervical cancer undergoing chemo-radiation in order to reduce rectal toxicity from external beam radiation as well as brachytherapy.

**Material and methods:**

Under transrectal sonographic guidance five patients with proven cervical cancer underwent hydro gel (20 cc) instillation into the tip of rectovaginal septum adherent to posterior part of the visible cervical tumor. Five days after this procedure all patients underwent T2 weighted transversal and sagittal MRI for brachytherapy planning. MRI protocol included T2 weighted fast spin echo (FSE) imaging in sagittal, coronal and para-axial orientation using an 1.5 Tesla MRI. Separation of anterior rectal wall and cervix was documented.

**Results:**

Hydrogel application was uneventful in all patients and no toxicity was reported. Separation ranged from 7 to 26 mm in width (median 10 mm). The length of the separation varied between 18 and 38 mm (median 32 mm). In all patients displacement was seen in the posterior vaginal fornix, and/or at the deepest part of uterine cervix depending on the extension of the cul-de-sac in correlation to the posterior wall of the uterus. In patients with bulky tumor and/or deep (vaginal) extend of peritoneal cavity tumour was seen mainly cranial from the rectovaginal space and therefore above the hydrogeI application. Only in the extra-peritoneal (lower) part of the cervix a good separation could be achieved between the rectum and cervix.

**Conclusion:**

Hydrgel instillation in patients with cervial cancer undergoing chemoradiation is safe and feasible. Because of the loose tissue of the cul-de-sac and its intra- and extraperitoneal part, hydrogel instillation of 20 cc did not result in a sufficient separation of the cervix from anterior wall.

## Introduction

Primary chemoradiation (RCTX) is the treatment of choice in patients with locally advanced and/or lymph node positive cervical carcinoma [1]. Paradigm shift from radiation to RCTX lead to an improvement with regard to local control as well as progression free and overall survival [2]. Analysis of patterns of recurrence showed that locoregional control remains critical [3]. Nevertheless RCTX can be associated with considerable acute and late gastrointestinal (GI) toxicity [1,4]. Reported grade 3 and 4 late GI toxicity is in the range of 4%–40% depending on target volumes and radiation techniques used [5-8]. However, possible improvement of local control due to dose escalation has to take GI and genitourinary (GU) toxicity into account.

Compared with 3D era IMRT based techniques could demonstrate lower rates of high grade toxicity [9-12]. Efforts have been made to better target definition for external beam radiation and dose escalation [13-15], dose prescription and application of brachytherapy, as well as simultaneous consideration of therapy related toxicity. Some proposals for dose sparing to the small bowel like treatment in prone position or use of bowel displacement systems did not gain acceptance in clinical routine [16,17].

Although external beam radiation (EBRT) as well as brachytherapy (BT) may lead to rectal complications only few data available concerning rectal toxicity in the treatment of cervical cancer patients. Their comparability is limited because of different treatment regimens, doses of EBRT and BT, dose prescriptions, application techniques and toxicity scoring systems. In cervical cancer patients cumulative overall and ≥2 grade rectal toxicity has been recorded in 12%–19% [18,19].

Injection of human collagen into the space between prostate and rectum in prostate cancer patients have shown decline of rectal dose by 25%–50% [20-24]. Therefore this new technique was thought to be an interesting option even for patients with cervical carcinoma undergoing EBRT and BT. A separation of at least 10–15 mm would be sufficient to achieve 80% rectal dose reduction [20]. Present study was initiated to proof feasibility of hydrogel application in patients with advanced cervical cancer in order to reduce rectal toxicity. To our best knowledge the present study is the first reporting of hydrogel application in patients with cervical cancer.

## Material and methods

**Figure 1:**
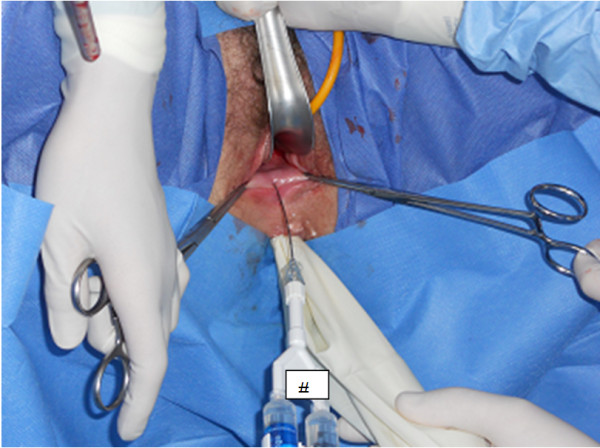
Grasters to the vaginal introitus in order to bring the rectovaginal septum under tension before application.

**Figure 2:**
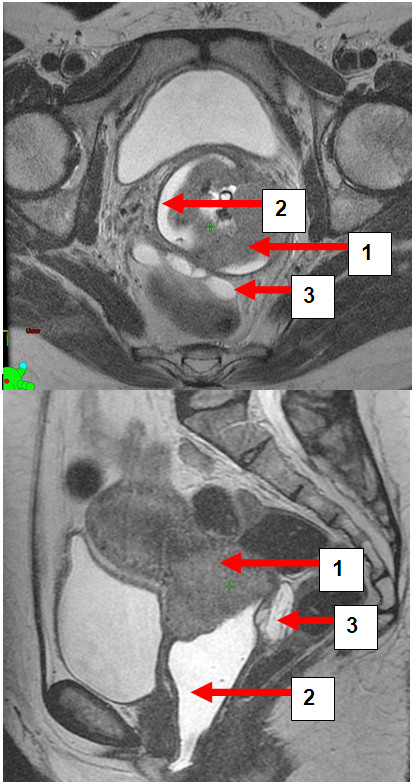
Instillation of the gel into the Douglas Space during general anaesthesia, # application set with the two components of the gel.

This pilot study comprises five patients with proven cervical cancer (age 31–69 years, BMI 20–49 kg/m^2^). FIGO stages were IB1 pN1 in one, IIB in two and IIIB in two patients, respectively. All patients underwent primary RCTX as previously described [25]. During third week of EBRT patients underwent implantation of Smit-Sleeve® applicator under general anaesthesia (Varian, Palo Alto, CA) for brachytherapy. During this procedure hydro gel installation was also performed. Written informed consent was given by all patients. The present pilot study was IRB approved. Patient was placed in with adducted legs placed on leg holders. The procedure started by filling the bladder with 300 cc isotonic saline solution. Under abdominal sonography external cervical os was identified, dilated and Smit Sleeve was inserted. After fixation of the applicator with non resorbable sutures graspers were placed at 05.00 and 07.00 o’clock at vaginal introitus. By pulling the graspers downwards rectovaginal septum was brought under tension before gel application (Figure 1). Now a vaginal sonography probe was inserted into the rectum. Under transrectal sonographic guide hydrogel applicator (SpaceOAR®, Augmenix, Waltham, MA) was placed up to the tip of rectovaginal septum adherent to posterior part of the visible cervical tumour. Injection was done bringing the two components of the gel (20 cc) together (Figure 2).

Five days after this procedure all patients underwent T2 weighted transversal and sagittal MRI for brachytherapy planning. MRI protocol included T2 weighted fast spin echo (FSE) imaging in sagittal, coronal and paraaxial orientation using an 1.5 Tesla GE Signa Excite (GE Healthcare, Fairfield, CO, USA).

## Results

**Figure 3:**
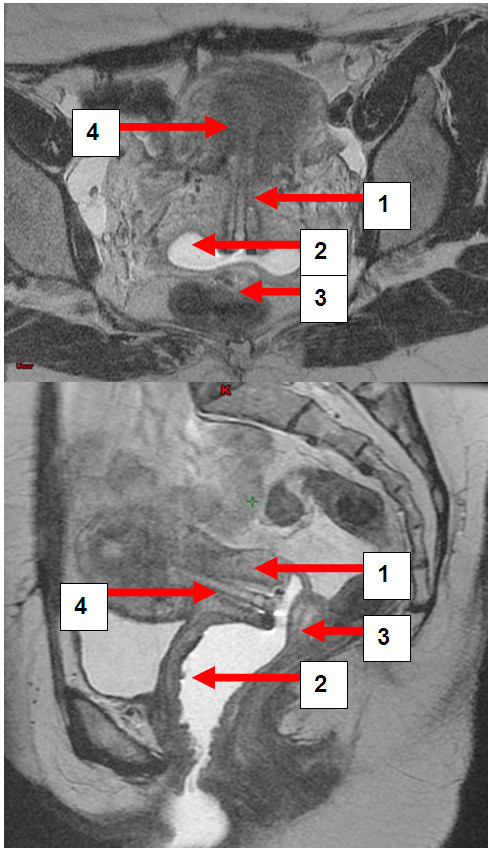
**a and b: Patient BW. **Transversal and sagittal pelvic MRI (T2-FSE) with cervical carcinoma (1) vaginal gel (2) and rectal separation (3).

**Figure 4:**
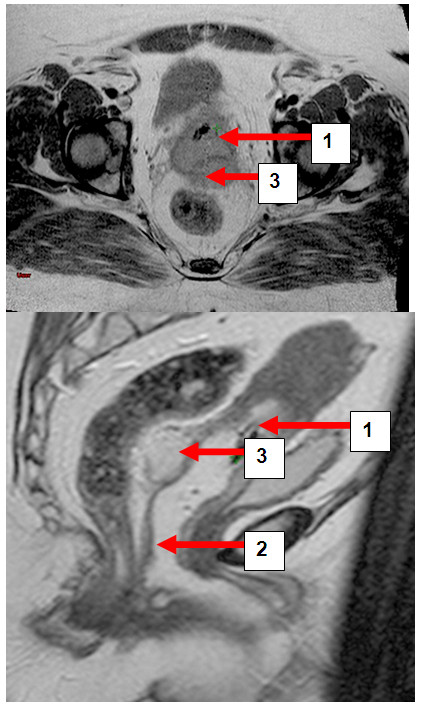
**a and b: Patient MB.** Transversal and sagittal pelvic MRI (T2-FSE) with the cervical carcinoma (1) vaginal gel (2), rectal separation (3) and afterloading applicator in situ (4).

**Figure 5:**
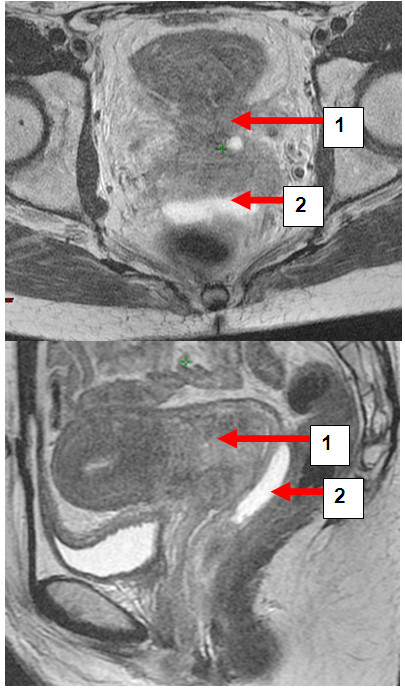
**a and b: Patient BR. **T2 weighted FSE in transversal and sagittal orientation with cervical carcinoma (1) vaginal gel (2) and rectal separation (3).

**Figure 6:**
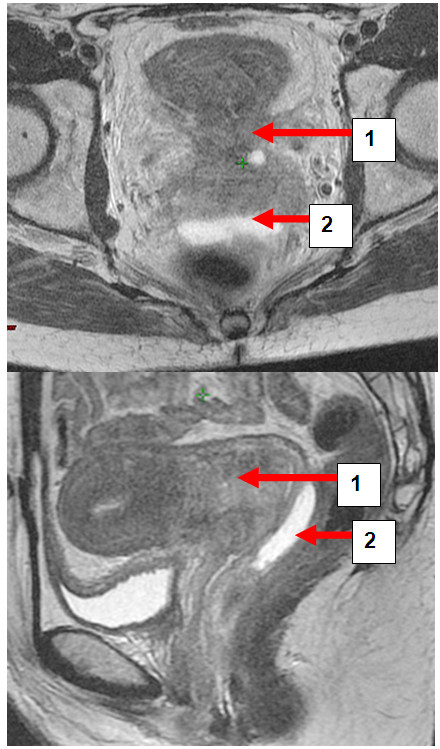
**a and b: Patient LC.** Transversal and sagittal pelvic MRI (T2-FSE) with cervical carcinoma (1) and rectal separation (2).

**Figure 7:**
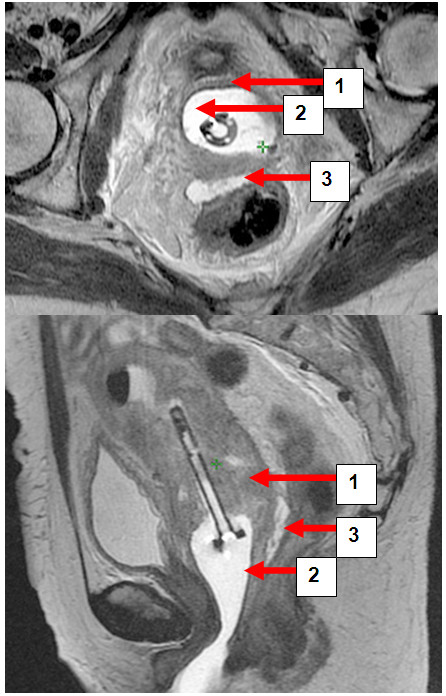
**a and b. Patient MB. **Transversal and sagittal pelvic MRI (T2-FSE) with the cervical carcinoma (1) vaginal gel (2) and rectal separation (3).

Hydrogel application was uneventful in all patients and no late toxicity was reported. On axial and sagittal T2-weighted MRI hydrogel was clearly visible as a hyperintense region between anterior rectal wall and posterior vaginal wall (Figures 1, 2, 3, 4 and 5). Extend of separation ranged from 7 to 26 mm in wideness (median 10 mm). The length of the separation varied between 18 and 38 mm (median 32 mm), (Table 1). In all patients displacement was seen only in the posterior vaginal fornix and/or deepest part of uterine cervix depending on the extension of the cul-de-sac in correlation to the posterior wall of uterus. In patients with bulky tumor or with deep vaginal extend of peritoneal cavity tumour was seen mainly cranial from the rectovaginal space and therefore above the hydrogeI application (Figures 3, 4, 6, 7). Only in the extra-peritoneal part of the cervix a good separation could be achieved between the rectum and cervix (Figure 5).

**Table 1:** Length and wideness of separation (cm)

**Pt. No.**	**Length of separation (cm)**	**Wideness of separation (cm)**
**1**	**3.15**	**1.30**
**2**	**1.79**	**1.02**
**3**	**3.49**	**2.61**
**4**	**2.61**	**0.71**
**5**	**3.77**	**1.04**

After completion of primary RCTX patients underwent gynaecologic examinations every 3 months. Additionally post radiation diagnostic curettage was done in order to exclude local recurrence at least 3 month after completion of therapy. In 4/5 patients no vital tumor cells were histologically found and they are free of disease 12 months after therapy. One patient had residual tumour in the cervix and synchronous pulmonary metastases.

## Discussion

Most data concerning rectal separation using hydrogel were published on prostate cancer patients. First cadaveric study has shown a decrease of rectal volume receiving 70 Gy significantly from 23% baseline value to 15%, 4% and 0% with 5 mm, 10 mm and 15 mm separation, respectively [20]. These data could be confirmed by other authors in clinical use during EBRT demonstrating a dose reduction to the anterior rectal wall by 50% [21]. This was associated with decreased risk for acute rectal toxicity compared with historical control [22] and improved quality of life [24]. Even for BT Prada et al. [22] could demonstrate that measured rectal dose for HDR boost was significantly lower by about 2 Gy in prostate cancer patients.

To our best knowledge, the present study is the first on cervical cancer patients with hydrogel instillation. Similarly to prostate cancer patients, the idea behind was to separate the rectum from the tumour infiltrated cervix uteri and thus reducing radiation dose to the anterior rectal wall during both external beam and brachytherapy planning. Alternative hydrogel distension could allow dose escalation without increasing risk for rectal toxicity.

There are two anatomical characteristis in female pelvis. In contrast to male anatomy with narrow and tense space between rectum and prostate (rectal fascia, Denonville fascia) rectovaginal septum is larger and contains more loose tissue. More volume of hydrogel would be necessary to develop entire rectovaginal space. However, median separation in our pilot study was 10.5 mm, which is in the range of the reported values from prostate cancer patients [20]. The length of separation has not been reported by other authors.

Second anatomical difference is the extent of the cul-de-sac in correlation to uterine/vaginal posterior wall which varies individually. In contrast to all studies on prostate that showed useful rectal separation, for cervical cancer we could only demonstrate a good separation for the extraperitoneal part of the cervix and upper vagina but no distension in the peritoneal part. This fact limits the value of the method for brachytherapy applications as well as EBRT. If by use of more hydrogel (approximately 100-150c) peritoneal cavity could be elevated and therefore rectum as well as small bowel could be more separated, remains speculative. Just as by other authors there was no toxicity from the instillation procedure in our study [20-24].

## Conclusion

Because of different anatomy in the female pelvis injection of 20 cc hydrogel does not seem to be a useful tool for rectal separation and thus for rectal dose sparing in patients with cervical cancer.

## Competing interests

The author declare that they have no competing interests.

## Authors' contributions

SM: Data acquisition and Paper Writing; Revision and corresponding author, VB: Idea and Concept, FW: Patient Management, EB: Patient Management, CK: Surgery and Gel Instillation, Manuscript Revision**,** BG: MRI scans, Diagnostics, Manuscript Revision. All authors read and approved the final manuscript.
